# Cholangioscope-Assisted Endoscopic Retrograde Appendicitis Therapy for Gangrenous Appendicitis in an HIV-Positive Patient

**DOI:** 10.5152/tjg.2026.25607

**Published:** 2026-02-23

**Authors:** Qianlong Li

**Affiliations:** Department of Gastroenterology, Suining Central Hospital, Sichuan, China

Dear Editor,

The traditional treatment for complex appendicitis primarily relies on surgery. However, for specific patient groups, including those with surgical intolerance, contraindications to surgery, compromised immunity, bloodborne infectious diseases, or pregnancy, surgical intervention may be associated with higher risks and thus may not represent the optimal first-line option.^1^ In recent years, cholangioscope-assisted endoscopic retrograde appendicitis therapy (ERAT) has been employed to treat acute, uncomplicated appendicitis.^2^^,^^3^ This technique enables direct visualization of the appendiceal cavity, facilitating precise treatment and thereby offering the potential for treating complex appendicitis in special populations.^4^ Here, we report for the first time an HIV-positive patient with gangrenous appendicitis who was successfully treated via cholangioscope-assisted ERAT.

A 50-year-old male presented with right lower abdominal pain that persisted for 24 hours. He had a fever, with his body temperature reaching 38.5℃. Notably, he had a history of HIV and was receiving combination therapy with tenofovir, emtricitabine, and dolutegravir. Physical examination revealed tenderness and rebound pain in the right lower quadrant of the abdomen. Laboratory examinations revealed a white blood cell count of 14.45 × 10^9/L, with 81.3% neutrophils. The level of C-reactive protein was measured at 23.8 mg/L. The Computed tomography (CT) showed significant thickening of the appendix with surrounding inflammatory exudate (Figure 1A). Given that the patient presented with fever and exhibited signs of peritonitis, anti-infective therapy with ceftriaxone (at a dose of 2 g once daily) and metronidazole (at a dose of 1 g once daily) was promptly administered immediately after admission. Due to the patient’s low immunity, the risk of surgical incision infection and intestinal fistula after appendectomy would be extremely high.^5^ Additionally, there was a risk of blood-borne infection during the surgery.^5^ Therefore, the surgeons recommended conservative treatment. However, the patient still had persistent high fever 48 hours after admission, despite the administration of antibiotics. After informed consent was obtained, a cholangioscope-assisted ERAT procedure was carried out under general anesthesia. The bowel preparation was performed with polyethylene glycol 4 hours before ERAT. The colonoscope, equipped with a short transparent cap, reached the appendiceal orifice. Congestion, edema, and pus adhering to the appendiceal orifice were observed. The cholangioscope was subsequently inserted through the forceps channel of the colonoscope and smoothly entered the appendiceal cavity. Under direct visual inspection with a cholangioscope, the appendiceal mucosa appeared dark brown, with a large number of pus-like substances adhering to it (Figure 1B), and the mucosa showed signs of stripping. Given the possibility of perforation, the pressure of the water-injection pump was decreased, and a large amount of pus was flushed out with normal saline. The appendix cavity was cleansed, and then, a plastic biliary stent was placed (Figure 1C, Figure 1D). Stent placement was performed using the following approach: After withdrawing the cholangioscope, the guidewire was left in the appendiceal cavity. Subsequently, an 8.5 Fr-7 cm stent was gently advanced along the guidewire into the appendiceal cavity. Immediately following the procedure, the patient experienced transient abdominal pain and fever. A post-procedure CT revealed a small amount of free gas around the appendix, and local minor perforation was suspected. Nevertheless, with the administration of antibiotics, the symptoms were completely alleviated 2 hours after the procedure. The patient had no recurrence of fever within 3 days after ERAT, and infection markers returned to normal. After 4 months of follow-up, the patient did not experience a recurrence of appendicitis. The CT indicated that the appendix had returned to normal and that the stent was in the proper position. A normal appendix opening was visualized, and the stent was removed under a colonoscope.

To present knowledge, this is the first report of cholangioscope-assisted ERAT being utilized in the treatment of acute gangrenous appendicitis in an HIV-positive patient. The patient exhibited unique clinical characteristics: Firstly, HIV infection-induced immunocompromise rendered the patient susceptible to postoperative infectious complications following conventional surgical appendectomy. Secondly, conservative treatment failed to alleviate the progressive inflammation of gangrenous appendicitis. With the advancement of endoscopic techniques and instruments, several studies have reported the successful application of ERAT in the management of certain complex appendicitis cases, such as periappendiceal abscess and localized perforated appendicitis.^6^^,^^7^ Cholangioscope-assisted ERAT enables direct visualization of the appendix cavity, thereby enhancing safety while performing procedures such as stenosis dilation, pus irrigation, and stone extraction, while simultaneously avoiding radiation exposure to medical personnel. In this case, direct visualization via cholangioscope confirmed the manifestation of gangrenous appendicitis. Sufficient irrigation, drainage, and decompression effectively avoided the high risk of surgical infection in the HIV patient with complex appendicitis, achieving satisfactory therapeutic outcomes. Cholangioscope-assisted ERAT may be an alternative treatment for patients with acute complex appendicitis complicated with immune deficiency. However, the case report has limitations, and further validation through more randomized controlled trials is required.

## Figures and Tables

**Figure 1.**
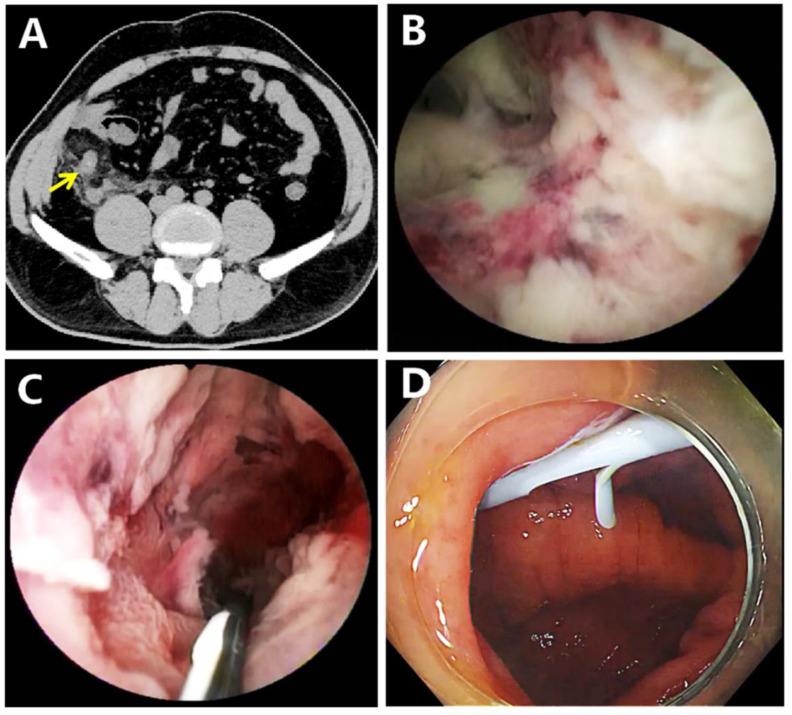
(A) The computed tomography revealed that the appendix was significantly thickened, along with peripheral inflammatory exudation (yellow arrow). (B) Cholangioscope showed that the appendiceal mucosa appeared dark brown. (C) A guidewire was implanted into the lumen of the appendix. (D) A plastic biliary stent was placed.

## Data Availability

The data that support the findings of this study are available on request from the corresponding author.
